# High-resolution haplotype block structure in the cattle genome

**DOI:** 10.1186/1471-2156-10-19

**Published:** 2009-04-24

**Authors:** Rafael Villa-Angulo, Lakshmi K Matukumalli, Clare A Gill, Jungwoo Choi, Curtis P Van Tassell, John J Grefenstette

**Affiliations:** 1 Department of Bioinformatics and Computational Biology, George Mason University, VA, USA; 2 Department of Animal Science, Texas A&M University, College Station, TX, USA; 3 Bovine Functional Genomics Laboratory, USDA-ARS, Beltsville, MD, USA

## Abstract

**Background:**

The Bovine HapMap Consortium has generated assay panels to genotype ~30,000 single nucleotide polymorphisms (SNPs) from 501 animals sampled from 19 worldwide taurine and indicine breeds, plus two outgroup species (Anoa and Water Buffalo). Within the larger set of SNPs we targeted 101 high density regions spanning up to 7.6 Mb with an average density of approximately one SNP per 4 kb, and characterized the linkage disequilibrium (LD) and haplotype block structure within individual breeds and groups of breeds in relation to their geographic origin and use.

**Results:**

From the 101 targeted high-density regions on bovine chromosomes 6, 14, and 25, between 57 and 95% of the SNPs were informative in the individual breeds. The regions of high LD extend up to ~100 kb and the size of haplotype blocks ranges between 30 bases and 75 kb (10.3 kb average). On the scale from 1–100 kb the extent of LD and haplotype block structure in cattle has high similarity to humans. The estimation of effective population sizes over the previous 10,000 generations conforms to two main events in cattle history: the initiation of cattle domestication (~12,000 years ago), and the intensification of population isolation and current population bottleneck that breeds have experienced worldwide within the last ~700 years. Haplotype block density correlation, block boundary discordances, and haplotype sharing analyses were consistent in revealing unexpected similarities between some beef and dairy breeds, making them non-differentiable. Clustering techniques permitted grouping of breeds into different clades given their similarities and dissimilarities in genetic structure.

**Conclusion:**

This work presents the first high-resolution analysis of haplotype block structure in worldwide cattle samples. Several novel results were obtained. First, cattle and human share a high similarity in LD and haplotype block structure on the scale of 1–100 kb. Second, unexpected similarities in haplotype block structure between dairy and beef breeds make them non-differentiable. Finally, our findings suggest that ~30,000 uniformly distributed SNPs would be necessary to construct a complete genome LD map in *Bos taurus *breeds, and ~580,000 SNPs would be necessary to characterize the haplotype block structure across the complete cattle genome.

## Background

The rapid improvement in high-throughput single nucleotide polymorphism (SNP) discovery and genotyping technologies is making possible the availability of many thousands of SNP markers for genome-wide association studies [1-5]. High-resolution linkage disequilibrium (LD) maps and characterizations of haplotype block structure are being generated for different organisms, confirming that elucidating in the fine-scale the structure of LD at the population level is crucial for understanding the nature of the highly non-linear association between genes and phenotypic traits, such as complex diseases and quantitative trait loci (QTL) [6-8].

Initial studies in humans [9,10] demonstrated that, by investigating regions for evidence of recombination and LD patterns, it was possible to parse the human genome into haplotype blocks, and that those blocks shared just a few common haplotypes. This result provided impetus for the construction of LD and haplotype maps of the human genome. Furthermore, haplotype block structure appears to be conserved across mammals [11].

Recently, high resolution LD and haplotype block maps were generated for humans using a set of 3.1 million SNPs genotyped in 270 individuals from four geographically diverse populations [12]. Overall, 98.6% of the assembled genome is within 5 kb of the nearest polymorphic SNP. The analysis of these high-resolution data is helping to infer with great precision, information about population history, recombination and mutation rates, evidence of positive selection, and is providing invaluable information for gene-disease association studies [13].

An initial bovine study [14] reported characterization of haplotype blocks in Holstein-Friesian cattle using a 15 K SNP chip with an average intermarker spacing of 251.8 kb. Another study [15] reported haplotype block structure for 14 European and African cattle breeds using 1536 SNPs. This study had an average resolution of 311 kb intermarker distance and was focused mainly on chromosome 3. Recently, the Bovine HapMap Consortium [16] generated an assay of 30 K SNPs and genotyped 501 animals sampled from 19 worldwide taurine (*Bos taurus*) and indicine (*Bos indicus*) breeds, plus two outgroup species (Anoa and Water Buffalo). In this article we present the characterization of LD and haplotype block structure across 101 high-density targeted regions from the bovine HapMap data, spanning 7.6 Mb of the genome with an average intermarker distance of ~4 kb. The extent of LD is presented along with the estimation of ancestral population size for different generations. In a first level of analysis, haplotype block characterization allowed us to elucidate the breed-specific block structure and its variability compared with all other breeds. In a second level of analysis, haplotype block density correlation, haplotype block boundary comparison, and haplotype sharing between breeds and subgroups helped us to elucidate high-resolution similarities between breeds, and also permitted us to differentiate breeds by geographic separation versus those related by shared ancestry. Finally, breeds were clustered given computed genetic distances based on haplotype block analysis.

## Results and discussion

Using the filtered data set (see Methods section for Quality Control filters) from the Bovine HapMap Consortium [16] consisting of 31,857 markers from 487 animals sampled from 19 cattle breeds (see Additional file 1), we selected the three chromosomes having the highest number of SNP markers, BTA 6, 14, and 25, and performed an analysis of high-density regions on these chromosomes. High density regions were originally genotyped in chromosomes 6 and 14 based on evidence of QTL and chromosome 25 based on a lack of known QTL (see Methods section). The high-density regions were defined as non-overlapping genomic windows of 100 kb containing 10 or more markers and a maximum gap between markers of 20 kb. We identified 101 such high-density regions covering a total genomic distance of 10.1 Mb (see Additional file 2). The effective region (regions within markers) covered is 7.6 Mb and contains in total 1,981 markers with an average of one marker each ~4 kb. The following sections discuss the haplotype block structure of these 101 high-density regions.

### SNP allele frequencies across population samples in high-density regions

**Figure 1:**
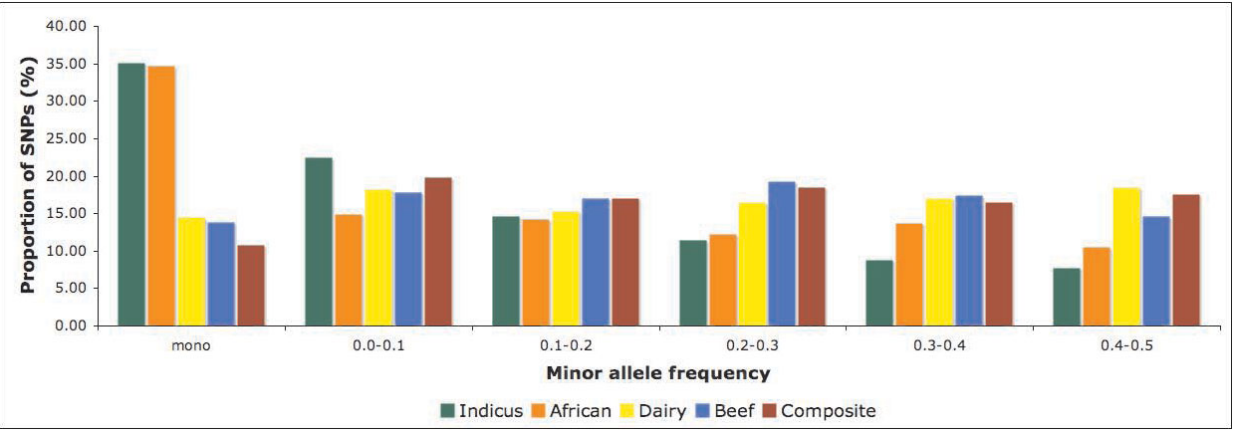
**MAF Distribution**. Average proportions of SNPs of various frequencies by cattle group in high-density regions (intervals' upper limit inclusive).

In order to investigate how informative the SNPs occurring in the targeted regions were, we computed the allele frequency distribution (Figure 1 presents the average by group, and Additional file 3 presents values by breed) and the average minor allele frequency (MAF) across all markers in the targeted regions (see Additional file 4). The breeds Nelore, N'Dama, and Gir exhibited the lowest proportion of polymorphic SNPs, between 57% and 62%, compared to the remaining breeds, which exhibited 77% to 95%. Thus a substantial fraction of loci in the targeted regions are informative for all breeds. Figure 1 presents all SNPs (including monomorphic and polymorphic SNPs) but for all subsequent analyses monomorphic SNPs were removed from the study.

In general, African and indicine breeds exhibited lower MAF values. It could be thought that this is due to an ascertainment bias in the SNP discovery because all targeted SNPs in this study were originally derived by comparison between a Hereford assembly and sequence reads from a series of bacterial artificial chromosomes (BACs) constructed from Holstein DNA. However, analysis of variation from among the major cattle breeds free from SNP ascertainment bias demonstrated a higher genetic diversity in indicine compared to taurine breeds [16]. In the targeted regions, MAF values ranged from a maximum of 0.253 (Holstein) to 0.116 (Nelore), which is a difference of about 28% in the full scale of 0.0 to 0.5. The average decay in MAF between breeds was 1.51%. (see Additional file 4). Furthermore, we compared the proportion of polymorphic SNPs in the selected regions with the proportion of polymorphic SNPs in the entire HapMap data set and found a 20% higher proportion in the complete HapMap data than the selected regions.

### Extent of LD and estimation of effective population size

The 1,981 SNPs in the high-density regions were used to evaluate the extent of pairwise LD as a function of physical distance. The complete set of SNPs (31,857) was used to estimate the effective population size in the previous 10,000 generations for each breed. A pair of haplotypes was inferred for each sample using the software fastPHASE version 1.2.3 [17], which provided imputed haplotypes for missing genotypes where necessary.

**Figure 2:**
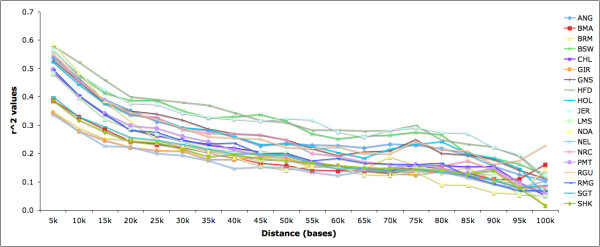
**Linkage Disequilibrium**. LD shows a rapid decline, such that r^2 ^averages ~0.1 at 100 kb. *r*^*2 *^values are averaged using bins of 5 kb.

The pairwise LD correlation coefficient *r*^2 ^was computed from the inferred haplotypes for all pairs of markers within each high-density region. Figure 2 shows the average of *r*^2 ^value using bins of 5 kb. Consistent with previous analyses in cattle [7,18], the decline of LD as a function of distance was rapid, such that *r*^2 ^averaged ~0.1 at 100 kb. Hereford, Jersey, and Brown Swiss had consistently higher *r*^2 ^values relative to the other breeds. In the case of Hereford and Jersey, this result is consistent with a lower resolution analysis (10 kb) previously performed using the same data [16]. In the case of Brown Swiss, the higher resolution inspection permitted us to elucidate its similarity in LD extent with the two previous breeds. As also shown previously [16], at the smaller distances N'Dama had the highest *r*^2 ^values while the *Bos indicus *breeds (Brahman, Nelore, and Gir) had the lowest values. In contrast, analyzing *r*^2 ^values at longer distances, Santa Gertrudis and Sheko were the breeds with the highest *r*^2 ^values while Angus and Beefmaster were the breeds with the smallest *r*^2 ^values. See Additional file 5 for the average *r*^2 ^value for each breed, computed as the mean *r*^2 ^value across all possible SNP pairs within each targeted region.

After adjusting *r*^2 ^for sample size error (see Methods section), we estimated the effective population size over the 10,000 previous generations (assuming a generation time of six to seven years [15]). This estimation was based on the observation that in a population with constant effective population size *N*, the approximate expectation of *r*^2 ^is: , where *N *is the effective population size 1/(2*c) *generations in the past, *E*(*r*^2^) is the average of *r*^2 ^values for all SNPs within a specified range, and *c *is the median of the range in Morgans (we assumed 1 cM ~1 Mb) [15,19-22].

**Figure 3:**
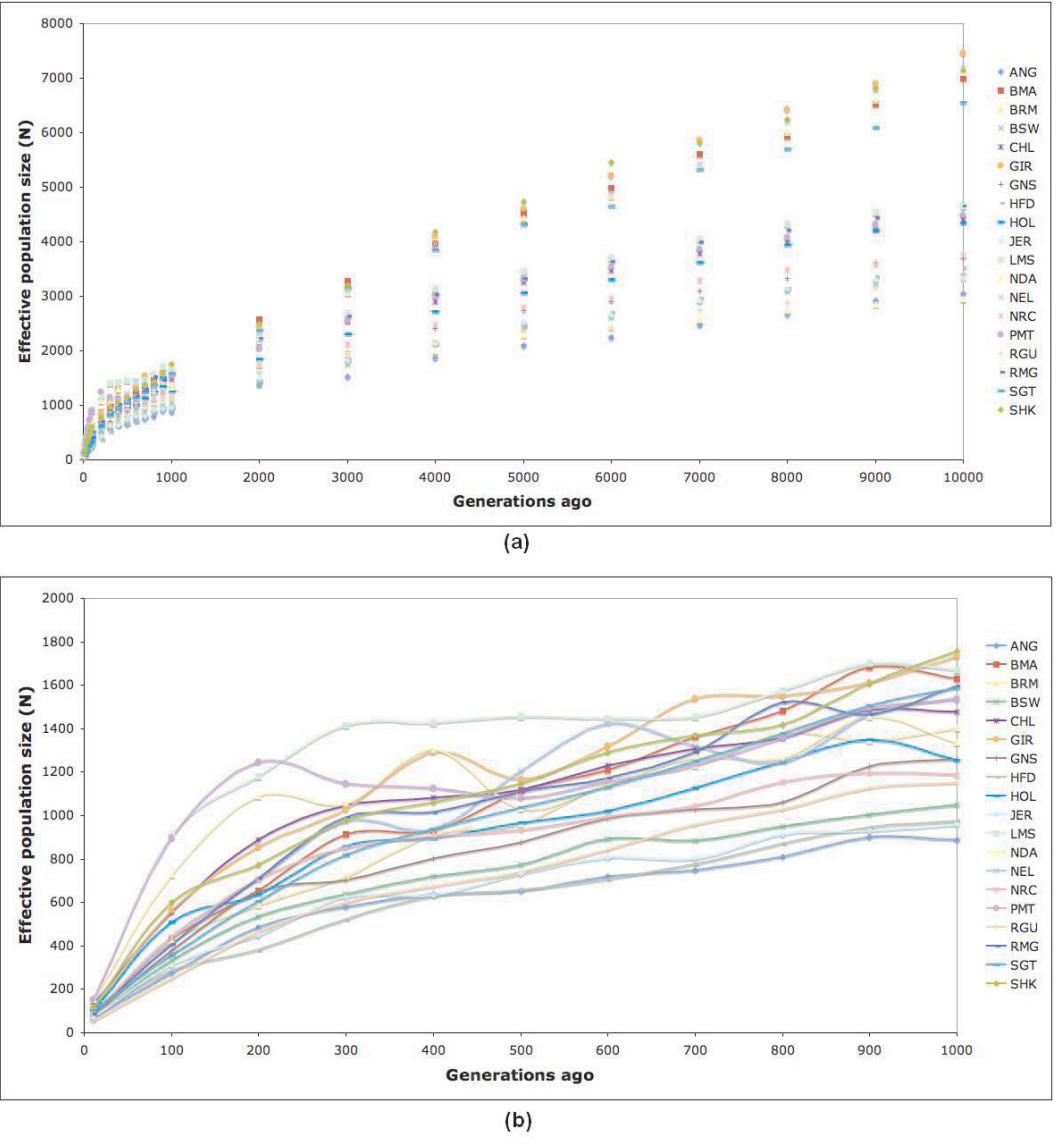
**Effective Population Size**. Estimated effective population size over previous generations suggest two distinct time points: the initiation of cattle domestication ~2,000 generations ago (a), and a population bottleneck in the most recent 100 generations (b).

The results show a persistent decline in effective population size through the period considered, but suggest two distinctive time points (Figure 3a). The first distinction is ~2,000 generations ago, at which time all population sizes seem to converge, compared to previous periods. The time associated with this convergence is approximately the early Neolithic period (~12,000 years ago) when domestication of cattle by humans began [16]. The second distinctive point is the most recent 100 generations, which show a sharp decline in population size (Figure 3b), suggesting that all breeds in this study are experiencing a population bottleneck. Two events may have contributed substantially to this reduction in effective population size: First, approximately 100 generations ago an intensification of population isolation was experienced principally in Europe, starting with the Great Famine of 1315–1322 followed by a series of large scale crises that struck Europe early in the 14th century, which caused significant reductions in the human population due to a great dearth of all victuals, and a dramatic reduction in livestock population sizes mainly due to a plague of murrain [15,23]. Second, the high selection pressure for specific traits and the use of artificial insemination have reduced dramatically the number of sires within the last ~50 years [21]. The estimated effective population size *N *for the most recent time point (10 generations ago) gave an average value of about 100 individuals across all populations. This result is similar to the average *N *of 116 reported in [16] in an analysis of these same samples. Additional file 6 presents the estimated effective population size for 10, 100, 1000, 5000, and 10000 generations ago for each breed in the study. We recognize that most breeds have originated more recently than 10,000 generations ago, but we assume that the estimates of effective population size in those cases should reflect the average historical population size of their ancestors.

### Haplotype block structure

Haplotype blocks based on *r*^2 ^were estimated using the definition from [24], discussed in the Methods section. Additional file 7 details the block characteristics for all breeds. In summary, the average maximum number of markers per block was 27.16. Across all breeds, 34.7% of the high-density regions were covered by haplotype blocks. We found that mean block size varied from 5.7 to 15.67 kb across breeds (with a mean block size of 10.3 kb over all breeds) and an average of 3.8 markers per block. These results are similar to those in a recent study of human haplotype blocks [25], which reported haplotype block sizes averaging 7.3, 13.2, and 16.3 kb in three human populations when analysing ten 500-kilobase regions with a density of one SNP per ~5 kb. The human data showed a marked decline in LD over the range of 1–100 kb, again similar to our observed decline in cattle LD from 0.6 to 0.1 over the range 1–100 kb.

From this and the results in the previous section, if we assume that the elucidated average of *r*^2 ^of ~0.1 in 100 kb, and that the haplotype block average size of ~10 kb with one informative SNP each ~5 kb are homogeneously distributed across the bovine genome, then, for constructing an LD map for association studies we should tag at least a SNP in each 100 kb. Therefore, we can estimate that it would be necessary to successfully assay at least 28,700 SNPs for a LD map for association studies. In the same way, it would be necessary to assay at least 574,000 SNPs to characterize the haplotype block structure across the entire bovine genome (assuming a bovine genome size of 2.87 Gb).

**Figure 4:**
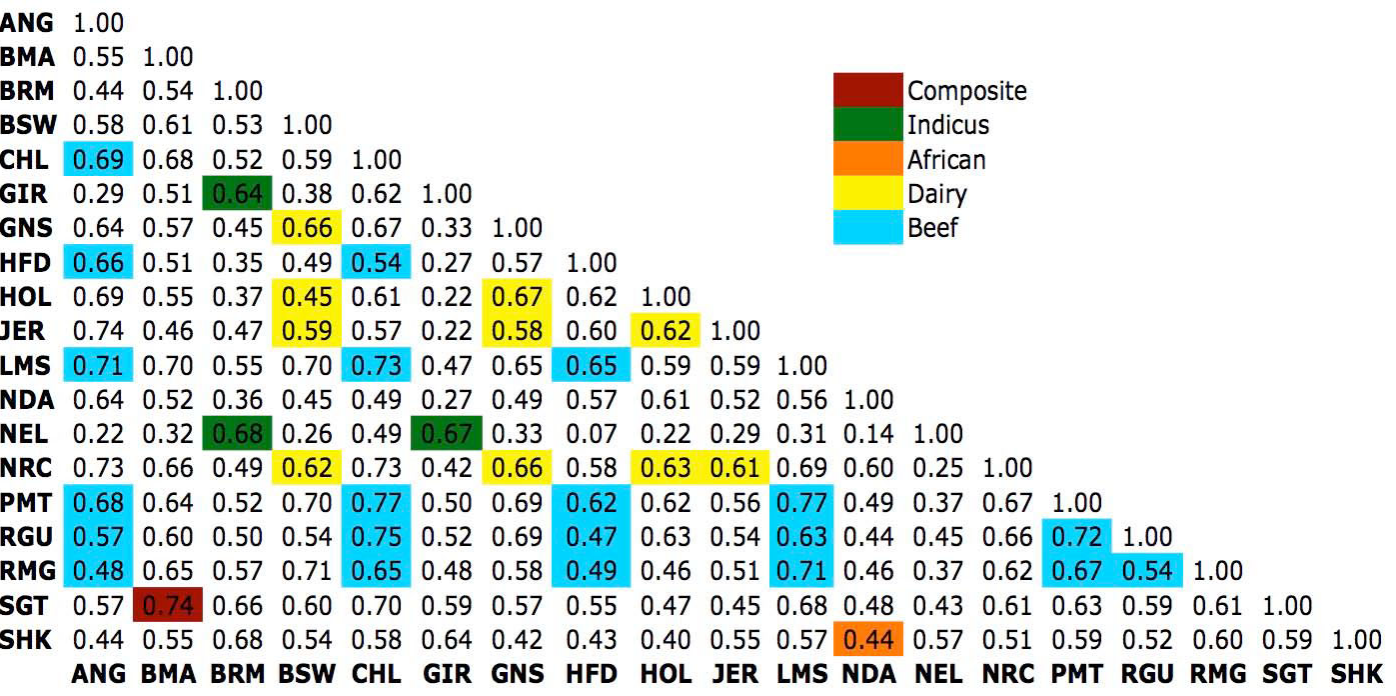
**Block density correlation**. Block density correlation across high-density regions shows the level of conservation in haplotype block structure among breeds from the same group.

**Figure 5:**
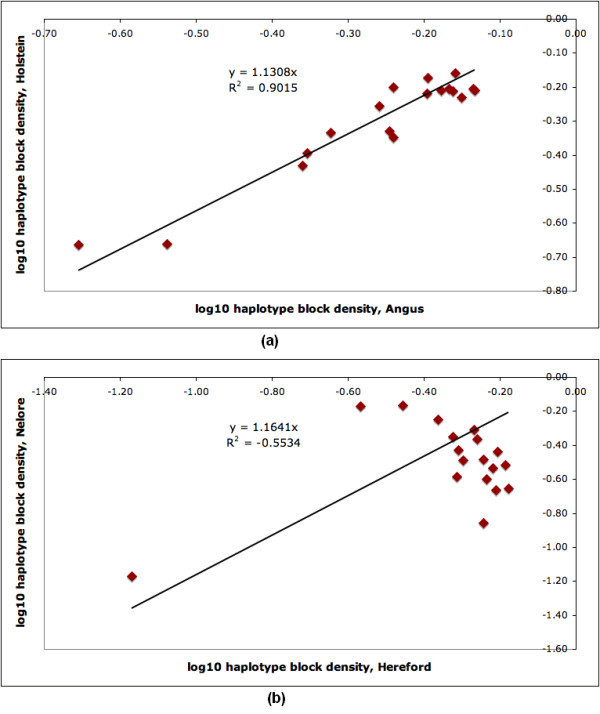
**Haplotype Block Density Correlation**. Comparison of Haplotype Block Densities between high-density regions of Holstein-a dairy breed- against Angus-a beef breed (both taurine) shows a high degree of correlation (a). Comparison of Nelore-an indicus breed against Hereford-a dairy breed (indicus against taurus) shows a low degree of correlation (b). The scatter plots show log_10 _of the amount of haplotype blocks for the same region in each breed pair.

To determine if the haplotype block structure in high-density regions is conserved among breeds, we counted the number of haplotype blocks occurring in each of the high-density regions for each breed, producing a 101-element *haplotype block density vector *for each breed. We calculated the correlation of the haplotype block density vectors between all pairs of breeds (Figure 4) and between several groups of breeds (Table 1). Figure 4 is color-coded to highlight the correlation between pairs of breeds in the same subgroup. The largest observed pair-wise correlations among breeds (0.77) occurred among Piedmontese, Charolais and Limousin (all three continental beef breeds). The smallest observed correlation was 0.07 between Hereford, a beef breed, and Nelore, an indicus breed. In general, indicus breeds showed small correlation with taurus breeds. For all subgroups, except African, the average within-group correlation was greater than the correlation with other subgroups. In the case of the African and composite breeds, the results may be biased by the sample size, having only two breeds from each subgroup. We observed a surprising degree of correlation between some subgroups, such as beef and dairy breeds. For example, Figure 5(a) presents the scatter plot of the density values (log_10 _values) of Holstein (a dairy breed) against Angus (a beef breed). Figure 5(b) shows a scatter plot for the lowest-correlation pair of breeds, Hereford and Nelore.

**Table 1:** Average haplotype block density correlations from all breeds within the group and outside the group.

Cattle group	Within group	Outside group
Beef	0.64	0.56
Dairy	0.61	0.54
African	0.44	0.51
Composite	0.74	0.57
Bos indicus	0.67	0.41

**Figure 6:**
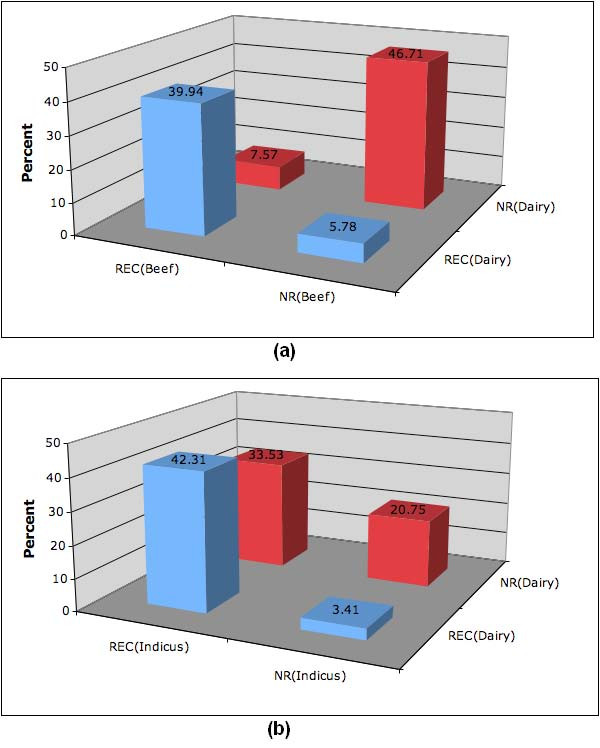
**Consistency in block boundaries**. Concordance and discordance of block assignments for adjacent SNP pairs (within SNP pair distance < 10 kb) in high-density regions. (a) dairy against beef breeds (both taurine), (b) dairy against indicus breeds (indicus against taurus).

We examined the consistency in block boundaries across breeds and subgroups by looking at adjacent pairs of SNPs in the high-density regions. Following the strategy of [10]: for each breed, if the SNP pair was inside a block, we termed it NR (having no evidence of recombination), and if the SNP pair was outside a block, we termed it REC (having evidence of recombination). Then, for a given pair of breeds or subgroups, a SNP pair was called *concordant *if the assignment was the same in both breeds (or subgroups) and *discordant *if the assignment disagreed. Results from comparing several groups of breeds are presented in Table 2. Figure 6(a) shows that approximately 13% of adjacent markers have discordant assignment in beef and dairy breeds when analyzed as subgroups. This level of discordance indicates a high degree of similarity in the fine-scale haplotype block structure between beef and dairy breeds, which suggests that a very detailed analysis of block discordance needs to be performed in order to differentiate between these two subgroups. On the other hand, Figure 6(b) shows that approximately 37% of marker pairs have discordant assignment when comparing the dairy subgroup against the indicus subgroup. This level of discordance indicates a fairly high degree of dissimilarity in haplotype structure between these two subgroups.

**Table 2:** Proportions of block boundary discordances and concordances among cattle subgroups

Comparison	Concordant	Concordant	Discordant	Discordant
	NR (%)	REC (%)	NR – REC (%)	REC – NR (%)
Beef vs Dairy	46.71	39.94	5.78	7.57
Beef vs Indicus	20.92	44.28	31.56	3.24
Beef vs Composite	37.11	42.31	15.38	5.20
Beef vs African	27.57	42.83	24.91	4.68
Dairy vs Indicus	20.75	42.31	33.53	3.41
Dairy vs Composite	36.47	39.88	17.80	5.84
Dairy vs African	26.94	40.40	27.34	5.32
Indicus vs Composite	19.88	53.41	4.28	22.43
Indicus vd African	17.23	60.81	6.94	15.03
Composite vs African	24.45	49.88	17.86	7.80

### Haplotype sharing

**Figure 7:**
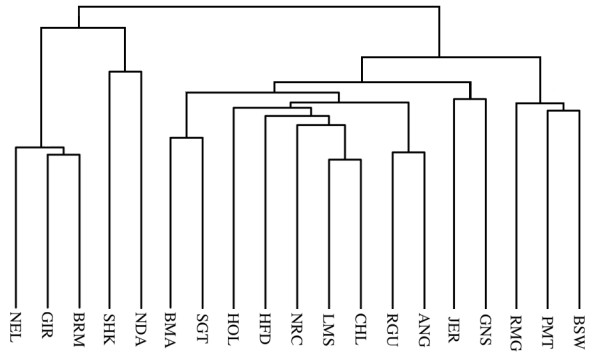
**Dendogram Based on Haplotype Sharing Distance**. Dendrogram based on genetic distance calculated from haplotype sharing.

We examined the multi-marker haplotypes associated within the high-density regions to provide further insight into relationships among breeds. The proportion of shared haplotypes provided another measure of similarity between different subpopulations. Table 3 shows the normalized proportion of shared haplotypes, averaged over all high-density regions, between various clusters of breeds. The most dramatic dissimilarity, as expected, is between all taurine and indicine populations. Figure 7 shows a dendrogram based on using the proportion of shared haplotypes within the high-density regions as a distance measure for clustering breeds. The dendrogram shows a clear differentiation for breeds of African origin (N'Dama and Sheko), for *Bos taurus/Bos indicus *composite (Beefmaster and Santa Gertrudis), and for indicus breeds (Gir, Nelore, and Brahman).

**Table 3:** Normalized proportion of shared haplotypes

	All Regions	BTA 6	BTA 14	BTA 25
ANG/HOL	0.47	0.59	0.40	0.48
Beef/Dairy	0.73	0.84	0.68	0.70
Taurus/Indicus	0.17	0.19	0.14	0.21

### Breed grouping

**Figure 8:**
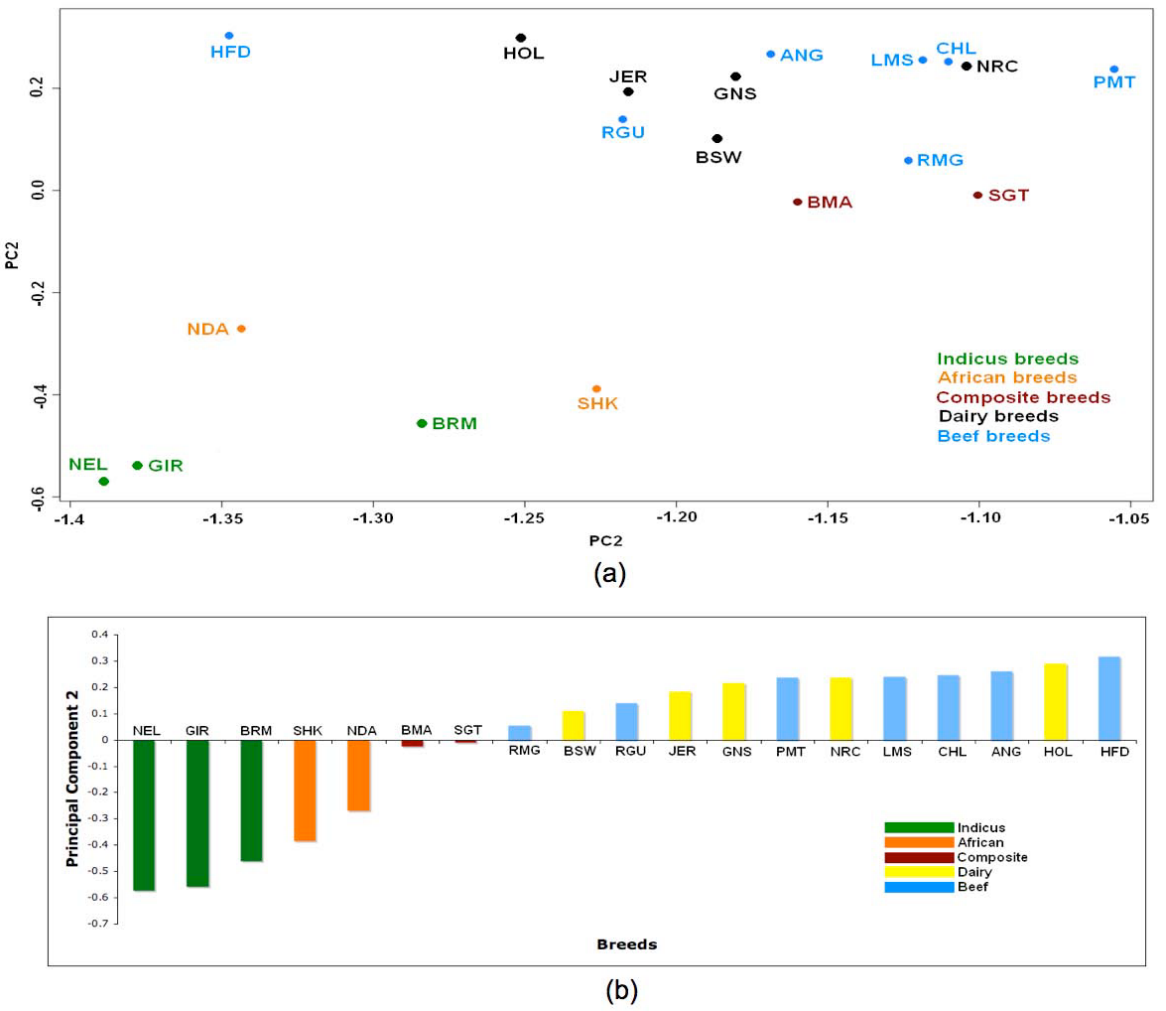
**Principal Component analysis**. Principal Component Analysis on block boundary discordance vectors shows how different breed subgroups as indicus, African, and Composite cluster together, but there is no clear separation between dairy and beef breeds. (a) Plot of PCA1 vs PCA2. (b) Plot of PCA2.

For each breed, we generated a *discordance vector *consisting of the percentage discordance found with all of the other breeds. Principal Component Analysis [26] was applied to these discordance vectors to provide another approach to clustering breeds by information based on haplotype block structure (Figure 8). For the subgroups we investigated, PCA shows the best cluster separation in the subspace defined by the second principal component, PC2 (Figure 8b). For PC2, indicus, African, and composite breeds have negative loadings, while the beef and dairy breeds, all *Bos taurus *breeds of British and European origin, have positive loadings. Santa Gertrudis and Beefmaster, known to be *Bos indicus/Bos taurus *composites, appear as intermediate between the two main subgroups. This result is consistent with previous PCA analysis performed directly on genotypes for the complete set of markers [16]. Both PCA analyses define a strong axis of variation separating taurine from indicine subgroups and placing composites as intermediates. However, the analyses differ in the principal component defining this relationship (PC1 for the genotype analysis, and PC2 for the block boundary discordances). In general, we consider that this analysis confirms that results obtained by analyzing the 1,981 SNPs in the selected high-density regions are consistent with the results obtained by analyzing all of the initial 30 k SNPs. We further observed that dairy and beef breeds cannot be clearly differentiated from this analysis. This result supports the hypothesis stated in the previous analysis [16] that historic geographic ancestry plays a stronger role in explaining genotypic variation (and haplotype block structure) in cattle than does their more recent selection into breeds with specific agriculture functions.

## Conclusion

In this work we present a high-resolution characterization of haplotype block structure in cattle. The analysis was performed on 101 targeted genomic regions spanning 7.6 Mb with an average density of one SNP each ~4 kb, sampled from 19 worldwide breeds. We studied LD and elucidated the block structure for each specific breed. Consistent with previous analyses in cattle, and in high agreement with observation in humans, we observed that LD declines rapidly, such that *r*^2 ^averages ~0.1 at 100 kb, and haplotype blocks exhibit an overall mean size of 10.3 kb (varying from 5.7 kb to 15.57 kb across all breeds) with an average of 3.8 markers per block. Estimation of effective population size in previous generations reflects the period of domestication ~12,000 years ago, as well as the current population bottleneck that breeds have experienced worldwide (last ~700 years) as a result of population isolation and selective breeding. In addition, an analysis of block density correlations, block boundary discordances, and haplotype sharing across all breeds and between subgroups were consistent in exhibiting a clear differentiation between indicus, African, and composite subgroups, but not between dairy and beef subgroups.

In summary, this work presents the first high-resolution analysis of haplotype block structure in worldwide cattle samples. First, novel results show that cattle and human share a high similarity in LD and haplotype block structure in the scale of 1–100 kb. Second, unexpected similarities in haplotype block structure between dairy and beef breeds make them non-differentiable. Finally, our results suggest that it would be necessary to successfully assay ~30,000 SNPs to construct an LD map for association studies, and ~580,000 SNPs to characterize the haplotype block structure across the entire bovine genome.

## Methods

### Animal samples and data description

The data used for this analysis correspond to the BTA4.0 assembly of the Bovine HapMap consortium database [16]. It includes genotypes from 501 animals on a set of 32,826 markers. Animals were sampled from 19 cattle breeds and two outgroups Anoa and Water Buffalo (see Additional file 1). All breeds belong to the taurus and indicus subspecies of *Bos taurus*, and represented several different geographical regions: N'Dama and Sheko are African breeds; Angus, Hereford, and Red Angus are British beef breeds; Charolais, Limousin, Piedmontese, and Romagnola are European beef breeds; Guernsey and Jersey are British dairy breeds; Brown Swiss, Holstein, and Norwegian Red are European dairy breeds; Brahman, Nelore, and Gir are indicus breeds; Beefmaster, and Santa Gertrudis are composites of taurine-indicine origin. Individuals were selected to be unrelated at least for 4–5 ancestral generations, with the exception of 44 trios of sire, dam and offspring included to allow quality control of the data and to assist in the determination of allelic phase relationships. The DNA samples were taken from whole blood or cryopreserved semen.

### Quality Control filters

To ensure the overall quality of samples and a consistent set of genotypes, QC filters were applied to the initial data (see [16]). The filters included removal of all genotypes that had >20% missing genotypes, that violated Hardy-Weinberg frequency distribution, or that violated Mendelian inheritance. Data were also removed for all animals with genotype completeness <98%, for markers with estimated genotyping error >5% and at least one breed out of Hardy-Weinberg equilibrium, as well as markers that were monomorphic for all breeds, markers with minor allele frequency <0.05 among all breeds, markers containing >2 discordant trios, and markers assigned to unknown chromosome. After this QC procedure, the data set contained 31,857 markers from 487 animals, and excluded Anoa and Water Buffalo.

In addition to previous QC filters, we removed monomorphic SNPs breed by breed in order to avoid the analysis of uninformative data.

### Selection of high-density regions

In order to facilitate the study of haplotypes extended over multiple markers, we focused on the regions of the bovine genome that had the highest density of markers in the HapMap data set. We focused exclusively on chromosomes 6, 14, and 25, which were selected for additional genotyping due to the presence of known QTL of interest in chromosomes 6 and 14, and the absence of known QTL on chromosome 25.

Chromosome 25 therefore served as a control for studies focusing on high-density regions. For this study, we defined high-density regions as non-overlapping genomic windows of 100 kb containing 10 or more markers and a maximum gap between markers of 20 kb. This definition identified 101 high-density regions contained a total of 1,981 markers, yielding an average density of 19.61 markers per region. The average distance between adjacent high-density regions on the same chromosome was 1.46 Mb, but they were not evenly spaced. There were 31 instances in which two adjacent high-density regions were contiguous on the chromosome.

### LD measure

A pair of haplotypes was estimated for each animal in the sample using fastPHASE Version 1.2.3 [17]. This software implements an Expectation-Maximization strategy for estimating missing genotypes and for reconstructing haplotypes from unphased SNP genotypes data of unrelated individuals. The LD measure adopted here was the squared correlation coefficient between SNP pairs (*r*^2^), computed as:



where, *p*_1 _and *p*_2 _are the minor and major allele frequencies in SNP 1 respectively, *q*_1 _and *q*_2 _are the minor and major allele frequencies in SNP 2 respectively, and *p*_11 _is the frequency of observing both minor alleles in the same individual across all population.

### Effective population size estimation

A correction for sampling error was made to all computed *r*^2 ^values as:



where *n *is the number of sampled haplotypes [18]. The effective population size was then estimated using the approximate expectation of *r*^2^:



where *N *is the effective population size 1/(2*c) *generations in the past, *E*(*r*^2^) is the average of *r*^2 ^values for all SNPs within a specified range, and *c *is the median of the range in Morgans [19-22]. To compute *N *for each breed, the number of previous generations was first selected. Then, *c *was computed in Morgans and taken as the median of the range (using a range of 10 kb and an approximation of 1 cM ≈ 1 Mb). The adjusted *r*^2 ^values were averaged for all SNP pairs within the range across all 29 autosomal chromosomes. We estimated *N *for 10 to 10,000 previous generations by using the complete set of SNPs (31,857 SNPs) since the set comprising just targeted high-density regions only permitted the estimation from N for 5,000 to 10,000 previous generations.

### Haplotype block estimation

Haplotype blocks were defined by the following algorithm [24]: (i) Begin a block by selecting the pair of adjacent SNPs with the highest *r*^2 ^value (no less than *α *= 0.4); (ii) Repeatedly extend the block if the average *r*^2 ^value between an adjacent marker and current block members is at least *β *(= 0.3) and all the pairwise *r*^2 ^values within the block are at least *γ *(= 0.1).

For each breed, we estimated the haplotype blocks along with some statistics as follows: first, we counted the number of blocks, then we computed the percentage of region covered in blocks by dividing the total distance within blocks over the total effective distance comprised in the 101 targeted regions, then we counted the number of markers per block and the block size mean. Finally we estimated the 95% Confidence Interval (*α *= 0.95) for the block mean size, assuming that block size follows a normal distribution, as:



where  denotes the sample average mean size, *s *denotes the sample standard deviation, *n *denotes the sample size, and  denotes the  percentile of a *t *distribution with *n-1 *degrees of freedom [27].

### Comparing Haplotype Block Structure Across Breeds

To determine if the haplotype block structure in high-density regions is conserved among breeds, we counted the number of haplotype blocks occurring in each of the 101 high-density regions for each breed, producing a 101-element vector for each breed.

Following [11], we computed Pearson product moment correlation coefficient, *r*, between each pair of breeds using the formula:



where *i *and *j *represent two breeds, *k *represents a high density region, *x*_*i*, *k *_and *y*_*j*, *k *_represents the number of haplotype blocks found in region *k *for breeds *i *and *j *respectively, and  and  represents the mean number of haplotype blocks found across all regions for breeds *i *and *j *respectively.

In order to assess the consistency of block boundaries across breeds, we examined adjacent pairs of SNPs with intermarker distances up to 10 kb. For each breed, it was determined whether the pair was assigned to a single block or not. Then, for a given pair of breeds, a SNP pair was termed *concordant *if the assignment was the same in both breeds and *discordant *if the assignments disagreed [10]. We performed this analysis for all pairs of breeds. In addition, we computed concordances and discordances between beef and dairy *g*roups, and between dairy and indicus groups as well.

### Haplotype Sharing

We analyzed the degree of sharing among the 19 breeds of phased haplotypes extending over multiple markers in the 101 high density regions. Haplotypes were inferred for all sample animals using the fastPHASE program (version 1.2.3). Each high-density region defined a locus for the purpose of the analysis. Haplotype segments were defined as the highest-probability haplotypes inferred by fastPHASE for each animal at each locus. The proportion of shared haplotypes between two populations *P*_1 _and *P*_2 _at locus *k *was defined as



where *i *and *j *range over the individuals in populations *P*_1 _and *P*_2_, respectively, *S*_*a*_(*i*, *j*, *k*) is the number of shared haplotypes between individuals *i *and *j *at locus *k*, and *n*_1 _and *n*_2 _are the number of samples in *P*_1 _and *P*_2_. The raw proportions were normalized to take into account the proportion of shared haplotypes within each of the individual populations, as follows:



*S'*(*P*_1_, *P*_2_, *k*) has value 1.0 if the proportional of shared haplotypes between populations *P*_1 _and *P*_2 _at locus *k *is equal to the average of the proportional of shared haplotypes within the two populations *P*_1 _and *P*_2_. If *S'*(*P*_1_, *P*_2_, *k*) << 1.0, then the proportion of shared haplotypes between the two populations is much less than the average within the two populations.

### Clustering Breeds

#### Clustering based on Shared Haplotypes

The proportion of shared haplotypes was used as a distance measure for clustering breeds. The normalized distance between breed *P*_1 _and *P*_2 _was calculated using:



where *u *is the number of loci. This is related to common measurements for genetic distance between two individuals [28-30]. *D'*(*P*_1_, *P*_2_) has value 0 if breeds *P*_1 _and *P*_2 _share the same proportion of haplotypes as are shared by the individuals within each individual breed.

#### Clustering based on Principal Components Analysis

Vectors resulting from the computation of haplotype block boundary discordances for each breed compared to the remaining breeds were used to perform a Principal Component Analysis (PCA) and look for differentiation between cattle subgroups. We used R software to perform this analysis. The central idea of PCA is to reduce the dimensionality of a data set which consists of a large number of interrelated variables, while retaining as much as possible of the variation present in the data set. This is achieved by transforming a new set of variables, the principal components (PCs), which are uncorrelated, and which are ordered so that the first few retain most of the variation present in all the original variables [26].

Formally, PCA is defined as an orthogonal linear transformation that transforms the data to a new coordinate system such that the greatest variance by any projection of the data comes to lie on the first coordinate (called the first principal component), the second greatest variance on the second coordinate, and so on. PCA is theoretically the optimum transform for a given data in least square terms. The procedure for obtaining PCAs can be summarized as follows:

Given a vector **X**^**T **^of *n *dimensions, *X *= [*x*_1_, *x*_2_,..., *x*_*n*_]^*T*^, whose mean vector **M **and covariance **C **are described by:



Calculate the eigenvalues *λ*_1_, *λ*_2_,..., *λ*_n_, and the eigenvectors *P*_1_, *P*_2_,..., *P*_*n*_; arrange them according to their magnitude.



Select *d *eigenvectors to represent the *n *variables, *d *<*n*. Then the *P*_1_, *P*_2_,..., *P*_*d *_are called the principal components.

## Authors' contributions

RV developed the algorithms for the analysis in the paper and drafted the article. JJG supervised the analysis, developed the algorithms for allele sharing, and contributed to the writing of the article. LKM and CAG contributed to database development and the quality control filtering of SNPs. All authors contributed to the structure and editing of the article, and approved the final paper.

## Supplementary Material

Additional file 1 **Breeds and number of animals in the sample.** Click here for file

Additional file 2 **Structural details of the 101 high-density regions selected on chromosomes 6, 14 and 25.** Click here for file

Additional file 3 **MAF distribution**. Average proportions of SNPs of various frequencies by breed in high-density regions (intervals' upper limit inclusive). Click here for file

Additional file 4 **Average minor allele frequencies (MAF) per breed across the high density regions in this study.** Click here for file

Additional file 5 **Total average of r2 per breed across high density regions.** Click here for file

Additional file 6 **Effective population size for each breed, estimated from r^2^.** Click here for file

Additional file 7 **Haplotype block structure across high-density regions in all breeds.** Click here for file
