# Supplementary material for: High-resolution haplotype block structure in the cattle genome
Source: BMC Genet. 2009 Apr 24;10:19. doi: 10.1186/1471-2156-10-19 (PMC2684545; doi:10.1186/1471-2156-10-19)
Supplement: Additional file 1 — Breeds and number of animals in the sample. [file 1471-2156-10-19-S1.doc]

## Additional file 7: Breeds and number of animals in the sample.

| Breed | No. of animals | Breed | No. of animals |
| --- | --- | --- | --- |
| Charolais (CHL) | 24 | Jersey (JER) | 28 |
| Limousin (LMS) | 42 | Norwegian Red (NRC) | 25 |
| Piedmontese (PMT) | 24 | Gir (GIR) | 24 |
| Romagnola (RMG) | 24 | Nelore (NEL) | 24 |
| Hereford (HFD) | 27 | Brahman (BRM) | 25 |
| Angus (ANG) | 27 | Beefmaster (BMA) | 24 |
| Red Angus (RGU) | 12 | Santa Gertrudis (SGT) | 24 |
| Brown Swiss (BSW) | 24 | Sheko (SHK) | 20 |
| Guernsey (GNS) | 21 | N’ Dama (NDA) | 25 |
| Holstein (HOL) | 53 | Buffalo and Anoa | 2 each |
