# Supplementary material for: High-resolution haplotype block structure in the cattle genome
Source: BMC Genet. 2009 Apr 24;10:19. doi: 10.1186/1471-2156-10-19 (PMC2684545; doi:10.1186/1471-2156-10-19)
Supplement: Additional file 2 — Structural details of the 101 high-density regions selected on chromosomes 6, 14 and 25. [file 1471-2156-10-19-S2.doc]

## Additional file 2: Structural details of the 101 high-density regions selected on chromosomes 6, 14, and 25.

|  | BTA 6 | BTA 14 | BTA 25 | Summary |
| --- | --- | --- | --- | --- |
| High-density regions | 30 | 57 | 14 | 101 |
| Markers in regions | 545 | 1228 | 208 | 1,981 |
| Ave markers per region | 18.17 | 21.54 | 14.86 | 19.61 |
| Total distance | 3 Mb scanned  2,276,304  Effective | 5.7 Mb scanned  4,465,915  Effective | 1.4 Mb scanned  896,479  Effective | 10.1  7,638,698 |
| Max gap between  markers (kb) | 19.47 | 19.7 | 19.35 | 19.7 |
