# Supplementary material for: High-resolution haplotype block structure in the cattle genome
Source: BMC Genet. 2009 Apr 24;10:19. doi: 10.1186/1471-2156-10-19 (PMC2684545; doi:10.1186/1471-2156-10-19)
Supplement: Additional file 3 — MAF distribution. Average proportions of SNPs of various frequencies by breed in high-density regions (intervals' upper limit inclusive). [file 1471-2156-10-19-S3.doc]

## Additional file 1: MAF Distribution


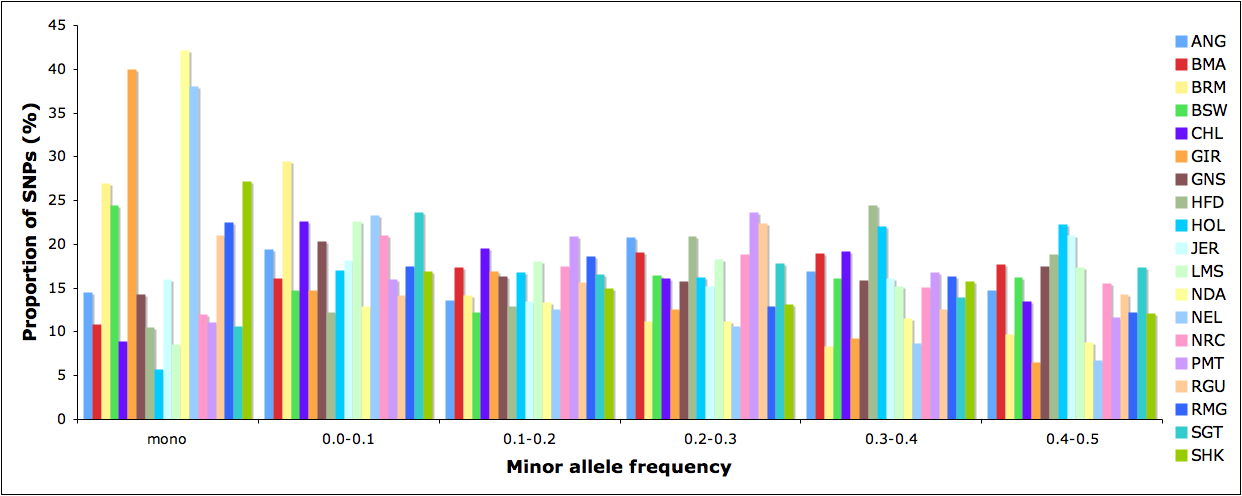


Average proportions of SNPs of various frequencies by breed in high-density regions (intervals’ upper limit inclusive).
