# Supplementary material for: High-resolution haplotype block structure in the cattle genome
Source: BMC Genet. 2009 Apr 24;10:19. doi: 10.1186/1471-2156-10-19 (PMC2684545; doi:10.1186/1471-2156-10-19)
Supplement: Additional file 4 — Average minor allele frequencies (MAF) per breed across the high density regions in this study. [file 1471-2156-10-19-S4.doc]

## Additional file 3: Average minor allele frequencies (MAF) per breed across the high density regions in the study.

| Breed | Average MAF | Value in the scale  0.0 – 0.5 (%) | Decay with respect to the previous breed (%) |
| --- | --- | --- | --- |
| Holstein (Dairy) | 0.253 | 50.6 | 0 |
| Hereford (Beef) | 0.250 | 50 | 0.6 |
| Beefmaster (Composite) | 0.227 | 45.5 | 4.5 |
| Jersey (Dairy) | 0.216 | 43.2 | 2.3 |
| Limousin (Beef) | 0.215 | 43 | 0.2 |
| Charolais (Beef) | 0.210 | 42 | 1 |
| Norwegian Red (Dairy) | 0.210 | 42 | 0 |
| Santa Gertrudis (Composite) | 0.210 | 42 | 0 |
| Piedmontese (Beef) | 0.209 | 41.8 | 0.2 |
| Guernsey (Dairy) | 0.208 | 41.6 | 0.2 |
| Angus (Beef) | 0.206 | 41.2 | 0.4 |
| Brown Swiss (Dairy) | 0.196 | 39.2 | 2 |
| Red Angus (Beef) | 0.193 | 38.6 | 0.6 |
| Romagnola (Beef) | 0.181 | 36.2 | 2.4 |
| Sheko (African) | 0.180 | 36 | 0.2 |
| Brahman (Indicus) | 0.140 | 28 | 8 |
| N’Dama (African) | 0.133 | 26.6 | 1.4 |
| Gir (Indicus) | 0.125 | 25 | 1.6 |
| Nelore (Indicus) | 0.116 | 23.2 | 1.8 |
