# Supplementary material for: High-resolution haplotype block structure in the cattle genome
Source: BMC Genet. 2009 Apr 24;10:19. doi: 10.1186/1471-2156-10-19 (PMC2684545; doi:10.1186/1471-2156-10-19)
Supplement: Additional file 5 — Total average of r2 per breed across high density regions. [file 1471-2156-10-19-S5.doc]

## Additional file 4: Total average of *r^2^* per breed across high density regions.

| Breed | r^2^ average | Breed | r^2^ average |
| --- | --- | --- | --- |
| Hereford | 0.397 | Romagnola | 0.283 |
| Jersey | 0.380 | Charolais | 0.278 |
| Brown Swiss | 0.377 | Limousin | 0.274 |
| Guernsey | 0.333 | Santa Gertrudis | 0.246 |
| Angus | 0.332 | Sheko | 0.236 |
| Red Angus | 0.330 | Beefmaster | 0.234 |
| Norwegian Red | 0.324 | Brahman | 0.230 |
| Holstein | 0.323 | Gir | 0.218 |
| N’Dama | 0.299 | Nelore | 0.204 |
| Piedmontese | 0.284 | Total r^2^ average | 0.294 |
