# Supplementary material for: High-resolution haplotype block structure in the cattle genome
Source: BMC Genet. 2009 Apr 24;10:19. doi: 10.1186/1471-2156-10-19 (PMC2684545; doi:10.1186/1471-2156-10-19)
Supplement: Additional file 6 — Effective population size for each breed, estimated from r2. [file 1471-2156-10-19-S6.doc]

## Additional file 5: Effective population size for each breed, estimated from *r^2^*.

| Generations Ago | 10 | 100 | 1000 | 5000 | 10000 |
| --- | --- | --- | --- | --- | --- |
| Angus | 64 | 275 | 890 | 2091 | 3042 |
| Beefmaster | 92 | 432 | 1629 | 4525 | 7008 |
| Brahman | 99 | 424 | 1402 | 4439 | 7095 |
| Brown Swiss | 68 | 335 | 1048 | 2430 | 3382 |
| Charolais | 130 | 554 | 1478 | 3263 | 4404 |
| Gir | 112 | 562 | 1732 | 4604 | 7460 |
| Guernsey | 76 | 378 | 1259 | 2737 | 3693 |
| Hereford | 83 | 288 | 974 | 2467 | 3520 |
| Holstein | 103 | 510 | 1256 | 3061 | 4350 |
| Jersey | 72 | 311 | 958 | 2523 | 3320 |
| Limousin | 154 | 911 | 1671 | 3456 | 4659 |
| N’Dama | 152 | 730 | 1336 | 2296 | 2946 |
| Nelore | 83 | 444 | 1545 | 4306 | 7199 |
| Norwegian Red | 89 | 437 | 1191 | 2808 | 3769 |
| Piedmontese | 151 | 898 | 1533 | 3310 | 4495 |
| Red Angus | 55 | 251 | 1151 | 2371 | 3303 |
| Romagnola | 87 | 408 | 1595 | 3321 | 4649 |
| Santa Gertrudis | 102 | 358 | 1587 | 4326 | 6562 |
| Sheko | 120 | 599 | 1759 | 4731 | 7145 |
