# Supplementary material for: High-resolution haplotype block structure in the cattle genome
Source: BMC Genet. 2009 Apr 24;10:19. doi: 10.1186/1471-2156-10-19 (PMC2684545; doi:10.1186/1471-2156-10-19)
Supplement: Additional file 7 — Haplotype block structure across high-density regions in all breeds. [file 1471-2156-10-19-S7.doc]

## Additional file 6: Haplotype block structure across high-density regions in all breeds.

| Breed | No of blocks | Regions in blocks (%) | Markers per block (max) | Markers per block (average) | Min block size (kb) | Max block size (kb) | Block size mean  (std) in kb | Block mean size 95 % Confidence Interval (min, max) in kb |
| --- | --- | --- | --- | --- | --- | --- | --- | --- |
| ANG | 282 | 41.61 | 38 | 4.21 | 0.25 | 68.08 | 11.28 (11.82) | 9.89 , 12.66 |
| BMA | 299 | 34.86 | 19 | 3.53 | 0.33 | 57.21 | 8.62 (8.86) | 7.61 , 9.63 |
| BRM | 233 | 19.61 | 16 | 2.98 | 0.25 | 30.38 | 6.72 (6.53) | 5.88 , 7.56 |
| BSW | 257 | 35.97 | 41 | 4.04 | 0.09 | 74.71 | 11.39 (12.61) | 9.84 , 12.94 |
| CHL | 302 | 40.7 | 20 | 4.01 | 0.07 | 67.26 | 10.48 (11.24) | 9.21 , 11.75 |
| GIR | 191 | 13.88 | 11 | 2.78 | 0.03 | 20.50 | 5.38 (4.84) | 4.69 , 6.07 |
| GNS | 289 | 39.71 | 16 | 4.08 | 0.51 | 54.28 | 10.79 (11.18) | 9.50 , 12.08 |
| HFD | 280 | 54.3 | 41 | 5.01 | 0.65 | 70.64 | 14.13 (13.35) | 12.56 , 15.70 |
| HOL | 307 | 47.46 | 47 | 4.36 | 0.38 | 63.81 | 11.45 (11.49) | 10.16 , 12.74 |
| JER | 302 | 40.77 | 41 | 4.01 | 0.31 | 65.65 | 11.11 (11.09) | 9.85 , 12.37 |
| LMS | 296 | 42.85 | 35 | 4.14 | 0.15 | 65.99 | 10.47 (11.52) | 9.15 , 11.79 |
| NDA | 211 | 22.15 | 14 | 3.29 | 0.30 | 37.21 | 8.39 (8.05) | 7.30 , 9,48 |
| NEL | 192 | 14.83 | 7 | 2.73 | 0.10 | 23.54 | 6.51 (5.36) | 5.75 , 7.27 |
| NRC | 298 | 43.61 | 21 | 4.12 | 0.53 | 70.29 | 12.28 (11.82) | 10.93 , 13.63 |
| PMT | 288 | 40.32 | 30 | 4.08 | 0.25 | 52.10 | 10.38 (9.71) | 9.25 , 11.50 |
| RGU | 264 | 38.23 | 32 | 4.07 | 1.14 | 58.20 | 11.58 (10.22) | 10.34 , 12.82 |
| RMG | 273 | 31.35 | 30 | 3.67 | 0.13 | 52.62 | 9.40 (9.01) | 8.33 , 10.47 |
| SGT | 298 | 34.58 | 24 | 3.54 | 0.04 | 49.85 | 8.37 (8.49) | 7.42 , 9.34 |
| SHK | 225 | 22.35 | 33 | 3.28 | 0.08 | 55.50 | 7.79 (8.93) | 6.62 , 8.96 |
